# Levels and health risk assessment of heavy metals in dried fish consumed in Bangladesh

**DOI:** 10.1038/s41598-021-93989-w

**Published:** 2021-07-19

**Authors:** Md. Refat Jahan Rakib, Y. N. Jolly, Christian Ebere Enyoh, Mayeen Uddin Khandaker, M. Belal Hossain, Shirin Akther, Abdullah Alsubaie, Abdulraheem S. A. Almalki, D. A. Bradley

**Affiliations:** 1 Department of Fisheries and Marine Science, Faculty of Science, Noakhali Science and Technology University, Noakhali, Bangladesh; 2 Atmospheric and Environmental Chemistry Laboratory, Chemistry Division Atomic Energy Centre , Dhaka, 1000 Bangladesh; 3 Group Research in Analytical Chemistry, Environment and Climate Change (GRACE&CC), Department of Chemistry, Imo State University (IMSU), PMB 2000, Owerri, Imo State Nigeria; 4 Center for Applied Physics and Radiation Technologies, School of Engineering and Technology, Sunway University, Bandar Sunway, 47500 Selangor, Malaysia; 5 Department of Physics, College of Khurma, Taif University, P.O. Box 11099, Taif, 21944 Saudi Arabia; 6 Department of Chemistry, Faculty of Science, Taif University, Taif, 21974 Saudi Arabia; 7 Department of Physics, University of Surrey, Guildford, GU2 7XH UK; 8 School of Engineering and Built Environment, Griffith University, 170 Kessels Road, Queensland, Australia

**Keywords:** Biogeochemistry, Environmental sciences, Gastroenterology

## Abstract

As a cheap source of high-quality protein, healthy fats and essential nutrients, dried fish is a common item in the daily diet of the Bangladesh populace. In this study, ten types of widely consumed dried fish (*H. neherius, T. lepturu, P.chinensis, P. affinis, A. mola, P. microdon, I. megaloptera, C. dussumieri, L. calcarifer, and G. chapra*) were analyzed for Cr, Mn, Fe, Co, Cu, Zn, Se, Rb, Hg, Pb, Ni and As by using an Energy Dispersive X-ray Fluorescence (EDXRF) technique. The concentration of the studied metals was found in the order Fe > Zn > Hg > Cu > Se > Cr > Mn > Co > Rb > Pb, while As and Ni were below the limit of detection. All fish species showed moderate to high pollution, where the species *H. Neherius* and *P. Chinensis* are the most and least polluted ones, respectively. The probable source of contamination is the leaching from the drying pans into the fish samples, atmospheric deposition, anthropogenic contamination, etc. of the water body where these fish were harvested. The calculated hazard index for the general population was below the maximum limiting value (i.e., < 1) except for Hg to children. The carcinogenic risk showed values lower than the acceptable limit for cancer risks (10^–6^ to 10^–4^). Periodic monitoring of trace metals in the aquatic organisms along with fish is recommended to avoid any unexpected health hazards caused by the toxic heavy metals via fish consumption.

## Introduction

Fish is one of the major and easily available protein sources for the people of Bangladesh, contributing about 60% of the total animal protein demand. According to a report of the Bangladesh Bureau of Statistics^1^, the per capita fish consumption in Bangladesh reaches 62.58 g per day, being greater than the daily protein demand of 60 g. Being an agro-based country, the fisheries sector is playing a significant role in the national economy of Bangladesh. Also, serving as the primary source of animal protein, this sector offers employment opportunities, earning of foreign currency, socio-economic development, etc^2^. Particularly, this sector contributes around 24.41% to the agricultural GDP and 3.61% to the national GDP of Bangladesh^3^. Within the last four decades, the total fish production in Bangladesh has increased significantly (by some sixfold), and it is now expected to reach 4.6 million tons by 2020–2021^2^. Based on a recent report of the Food and Agricultural Organization (FAO)^4^, Bangladesh stands at the 3rd largest position in terms of inland fish production, 5th in aquaculture production, and 11th in the marine fish production in the world. As Bangladesh is a riverine country and has a long coastline on the northern littoral of the Bay of Bengal, varieties of fish are naturally found abundantly throughout the year. In addition to the favourable natural habitats in support of fish production, fish farming is also showing itself to be greatly popular to the local populace. As a result, a sufficient amount of fish is produced in the country, Bangladesh also getting global recognition as one of the largest fish producing countries in the world^2^.

However, fresh fish deteriorate rapidly unless they are preserved in some way. Among many other processes, drying is one of the methods of fish preservation, working via the removal of water content to manifestly inhibit the growth of microorganisms. Sun drying is one of the most widely used techniques for preservation of fish in Bangladesh. Horner^5^ addressed drying as a curing process for fish preservation, and thus far its practice has been greater than the other contemporary food preservation techniques. Consequently, a large amount of freshwater and marine fish are preserved by this process, both for domestic consumption as well as for export purposes. In fact, every year, a sizeable amount of sun-dried fish is exported to meet the demands of international markets. Dried marine fish are more popular than fresh water varieties as they can be stored and consumed throughout the year by the local populace. Further to be appreciated is that dried fish is a highly rich source of nutrition, comprising 80–85% protein.

While it is accordingly appreciated that dried fish represents a popular and nutritious delicacy globally, frequent consumption may just conceivably infer a risk to health, some preservatives containing particular elements that can build up to toxic levels in body stores within the body. Contamination of toxic heavy metals in fish is a common problem worldwide. Trace elements in fish may come from various sources available in the aquatic environment, including within the waterbody itself, stored in the sediment and/or the terrestrial environment, the dietary habit of the fish, the preservation process and handling etc^6^. Toxic heavy metals are understood to enter the aquatic food chain via both the dietary (direct consumption of water and biota) and non-dietary (uptake through absorbing epithelia in fish)^7^ routes, thus aquatic organisms accumulate metal concentration several fold greater than that of the surrounding medium, and become an important media to transfer toxic metal from one trophic level to another.

Many studies have pointed to adverse effects to human health that may occur through the consumption of fish contaminated with trace metals, and some known diseases have been associated with trace metals. For instance, mercury has been implicated in neurological effects, cadmium causes carcinogenic diseases, and lead is a neurotoxin that causes a behavioral deficit in vertebrates and can cause a decrease in survival, growth rate and learning. A metabolism level of 50 ppm in diet may cause reproductive effects in some predators, and a low dietary level of 0.1–0.5 ppm may lead to a learning deficit of some vertebrates^8^. Lead is also carcinogenic, it can also damage the nerve system and haematosis in humans while copper has been connected to anemia. Chromium has carcinogenic and ulcerative characteristics. Zinc is an essential element but at high concentration leads to lung diseases, gastroenteritis, fever, vomiting, muscular coordination problem and dehydration^9–12^. In the process of drying of fish, sun-dried included, the level of toxic elements will become more concentrated and in combination with atmospheric deposition during drying, the probabilistic health effect in consumption of those fish may increase many fold. While a number of studies have been conducted on toxic heavy metals in different edible fish species there are only limited studies on dried fish toxicity. As dried fish occupies a special place in the diet and in some societies consumed frequently, there is a need to determine the level of heavy metals in dried fishes, and assess the concomitant risk associated with their dietary intake. With this in mind, the main aims of this study are to determine the concentrations of selected heavy metals such as chromium (Cr), arsenic (As), lead (Pb), copper (Cu), manganese (Mn), and zinc (Zn) in the more largely consumed species of dried fish available in Bangladesh, and also to assess the associated risk to human health via their consumption, offering representation of several different habitats.

## Materials and methods

### Sample collection and preservation

**Figure 1:**
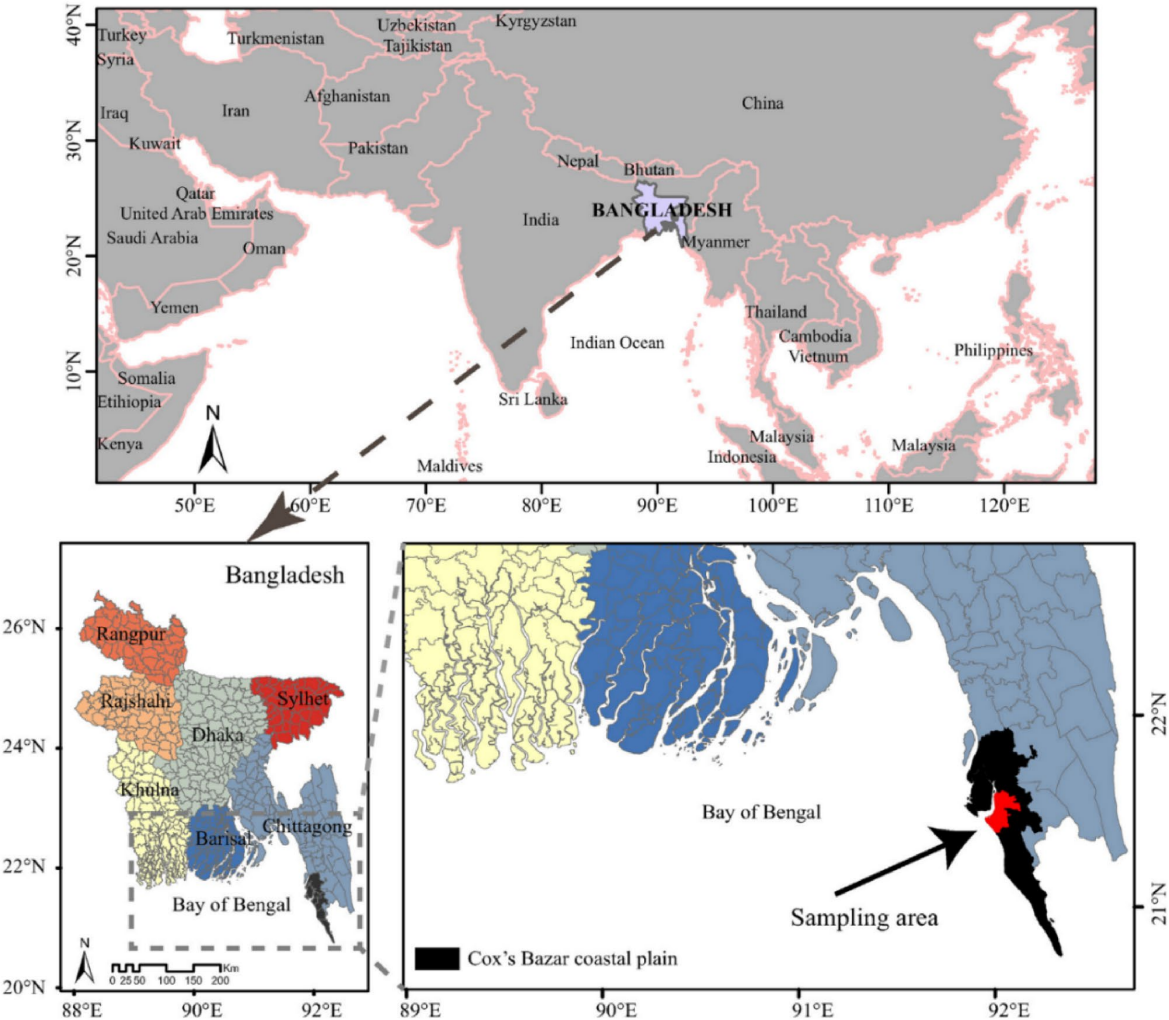
Sampling area in Bangladesh. Reprinted with permission from ref.^27^. Copyright, 2016, Elsevier.

Popularly consumed locally are ten different fish species (the local names being identified in brackets): *Harpodon neherius* (Loitya), *Trichiurus lepturus* (Chhuri), *Pampus chinensis* (Rupchanda), *Penaeus affinis* (Shrimp), *Amblypharyngodon mola* (Mola), *Panna microdon* (Poa fish), *Ilisha megaloptera* (Chowka faisha), *Coilia dussumieri* (Olua), *Lates calcarifer* (Coral fish), *Gudusia chapra* (Chapila). These were collected in triplicate, from different fish markets in Cox’s Bazar and Chittagong region of Bangladesh (Fig. 1). The collected fish were washed using tap water to remove mud or other foreign materials, then being wrapped in fresh polyethylene zipper bags and transported to the laboratory for further treatment. The edible portion (the muscular component of tissue) of each collected fish was separated and chopped into small pieces using stainless steel scissors. In advance of this, the scissors were cleaned with acetone, conducted a minimum of three times. Moreover, with the samples being in dried form no practical chance arises of leaching of elements from the scissors. Using an agate mortar and pestle the dried samples were then ground to fine powder, sieving being carried out using a well washed fine-mesh plastic sieve, with subsequent storage of the sieved powder in clean and dry airtight plastic vials inside a vacuum desiccator in readiness for analysis.

### Preparation of sample pellets and irradiation by EDXRF

In readiness for EDXRF analysis a ball milling procedure (Pulverisette 0: FRI_12007, FRITSCH GmbH, Germany) was applied in initial crushing of the dried fish species, and finally a fine homogeneous powder was obtained by grinding in an agate mortar and pestle facility. From 0.1 g of each powdered mass a pellet of 0.7 cm diameter and 1 mm thickness was prepared, applying 10ton pressure for approximately 3 min using a hydraulic press pellet maker (Specac, UK)^13^.

The pellets were loaded into an X-ray excitation chamber and irradiated using a 30 mCi Cd^109^ annular source for 1000 s. A Si(Li) detector of energy resolution 175.0 eV at 5.9 keV detected the characteristic X-rays, with data collected by a multichannel analyzer and transferred to the computer for storage, subsequent processing and evaluation of the net X-ray intensities. The software AXIL and PRO/QXAS (IAEA) were used for quantitative and qualitative elemental analysis^14^.

### Method validation

In any analytical technique, validation of the system/method is critical. Specifically, in use of the EDXRF technique a calibration curve needs to be obtained using a certified reference material (CRM) for validation of the entire process. With EDXRF representing a comparison technique, the main concern is that both the real sample and CRM must be of a similar matrix, hence producing identical sensitivity to effectively nullify matrix effects. For elemental analysis of fish samples, commercially available fish standard Tuna homogenates (IAEA-350) were used for the calibration, three pellets (Tuna-1, Tuna-2, Tuna-3) being prepared from the CRM. These were irradiated, the x-ray fluorescence spectrum obtained being used to construct the calibration curve in accordance with the step-by-step guideline of the software ‘AXIL’ installed within the computer.

Accuracy and precision of the constructed calibration curves were checked by analyzing the spectrum of DORM-2 (National Research Council, Canada) dogfish muscle (prepared and analyzed as for the real samples) following the procedure reported in Hasan et al.^15^. The precision was found to be 3–5%, accuracy of < 5%, and recovery percentage of the standard reference materials in the range 94–106% (Table 1). The EDXRF multielement measurement technique offers the capability to simultaneously detect a wide range of elements. Herein, detection of elements from K to Mo were available, the calibration curve allowing the concentrations of corresponding elements to be determined.

**Table 1:** Comparison between experimental results and certified values (mg kg^−1^, dry weight, DORM-2).

Element	Results obtained	Certified values	Relative error	CV (%)	Recovery (%)
As	16.9	18.0	5.89	5.84 × 10^–5^	93.7
Cr	30.07	34.70	9.34	3.8 × 10^–4^	86.7
Pb	0.071	0.065	−7.69	4.04 × 10^–4^	109.2
Hg	4.87	4.64	−4.56	4.08 × 10^–4^	105.0
Fe	139	142	4.19	5.27 × 10^–4^	97.9
Ni	20.9	19.4	−3.28	3.8 × 10^–4^	107.7
Zn	25.3	26.6	2.40	4.5 × 10^–4^	95.1
Cu	2.41	2.34	−2.87	3.24 × 10^–5^	103.0
Co	0.19	0.182	1.89	3.43 × 10^–4^	104.4

### Calculation of minimum detection limit (MDL) for fish matrix

The minimum detection limit (MDL) depends on the counting statistics of the measurement system and it is a statistical process. The MDL was obtained from the ratio of an element (in ppm) yielding an X-ray intensity equal to 3σ of the background under the peak in an interval equal to the FWHM of the peak and the sensitivity of the corresponding elements determined by using the calibration procedure^16, 17^, and calculated using relation (Eq. 1):

1 $$MDL(x) = {\text{ }}\frac{{3{\text{o}}^{\prime } {\text{counts of element /x/ in the sample}}}}{{\frac{{{\text{Counts}}}}{{{\text{ppm}}}}{\text{ of element}}/{\text{x}}/{\text{in the standard}}}}$$

where, $o'=\sqrt{\frac{Background}{Channel} \times FWHM}$ of the relevant element. Table 2 shows the calculated MDL of the studied elements in the fish matrix.

**Table 2:** Determined MDL for fish matrix.

Element	MDL (ppm)
Cr	0.27
Mn	0.28
Fe	0.27
Co	0.15
Cu	0.19
Zn	0.15
Se	0.21
Hg	0.12
Pb As Ni	0.03 0.41 0.24

### Statistical analysis

The determined metal concentrations values were tabulated and tested for normality and homogeneity of variance prior to the statistical analyses. One-way variance of analysis (ANOVA) was used to test for significant differences in metal levels among different dried fish species. All required analyses and graphical representations were performed using a combination of R Studio v.1.1.453, PAST^18^, and Prism 8.0 (GraphPad Software Inc., USA), and ArcGIS 10.6 software.

### Pollution and health risk assessment

The extent of contamination of dried fish by heavy metals was examined in terms of Metal Concentration Factors (MCF) and the Metal Pollution Index (MPI). Human health risks due to the determined metals in fish species via their consumption were calculated using several criteria/indices: Estimated Daily Intake (EDI) of metals, Target Hazard Quotient (THQ), Hazard Index (HI), and Cancer Risk (CR). Summary information on these hazard parameters is provided in Table 3.

**Table 3:** Description of the metal indices utilized in study of the present fish samples.

Indices	Purposes	Methods	References
Estimated daily intake (EDI)	The EDI was assessed using the metal concentrations in the studied fish and their consumption characteristics	$EDI=\frac{Cn\times IGr}{Bwt}$ (2) where, Cn represents the determined concentrations of heavy metal in the fish tissues (mg/kg dry-wt); IGr represents the ingestion rate (55.5 g/day for adults and 52.5 g/day for children); Bwt represents the body weight: (70 kg for adults and 15 kg for children)	^19–23^
Target hazard quotient (THQ)	THQ was calculated from the ratio of EDI to the oral reference dose (R_f_D)	THQs $=\frac{Ed\times Ep\times EDI}{At\times {R}_{f}D}$ ×10^–3^ (3) where, ED is the exposure duration (65 years), EP is exposure frequency (365 days/year); A_T_ is the average time for non-carcinogens (ED × EP). R_f_D (mg/person/day) of metals viz. Cr, Co, Cu, Zn, Hg, Pb and Mn is 0.003, 0.02, 0.04, 0.3, 0.0001, 0.004 and 0.046 respectively	^20, 23–28^
Hazard index (HI)	The HI can be used to assess the additive effects from various heavy metals taken via fish consumption	$\mathrm{HI}={\sum }_{i=k}^{n}THQ$ (4) where, HI > 1 refers for consumers experiencing significant health hazards due to non-carcinogenic metals exposure	^29–34^
Cancer risk(CR)	Carcinogenic risk describes the incremental probability of cancer in an individual over a lifetime, due to exposure to a substantial carcinogen	CR $=\frac{Ed\times Ep\times EDI\times CSF}{AT}$×10^–3^ (5) where, CSF is the oral slope factor of carcinogens (mg/kg/day) provided by USEPA (2010a, 2010b); CSF values are available only for Cr (0.003 mg/kg/day) and Pb (0.0085 mg/kg/day). The probability of the development of cancer in a consumer would be > 1 in 100,000, when CR values are above 10^−5^	^24, 35–44^
MCF and MPI	This index helps to assess the metal pollution level in the studied fish	$MCF=\frac{\mathrm{Concentration}\, {of}\, {a} \,{metal}\, {in}\, {fish}}{\mathrm{Concentration}\, {of}\, {a}\, {metal}\, {in}\, {water}}\times 100$ (6) MPI = (MCf_1_ × MCf_2_ × MCf_3_ × ……….. × MCf_n_)^1/n^ (7) where, MCF_1,2,..n_ represent the metal contamination factors for the different metals in the tissue sample of a certain species while n is the number of metals studied. In calculating the MCF the average values for each metal were used. Non-detected values were excluded from the calculation	^45–47^

## Results and discussion

### Metal concentration in fish species

This study reports the metal concentrations in dried fishes as dry weight basis. On the other hand, the permissible limits of various metal concentrations in foodstuffs, recommended by Food and Agriculture Organization (FAO)^48^, has been reported as wet weight basis. In order to make a fair comparison of the determined metal concentrations, the FAO data has been converted to dry weight using a simple relation (8):

8 $$Metal\, concentration\, in \,wet \,weight\left(\frac{mg}{kg}\right)=\frac{\left(100-\% \,of\, water\, in \,the \,sample\right) }{100}\times Concentration\, in \,dry \,weight$$

In general, fish contains some 70–84% water (moisture content), requiring to be dried (in case of dried fish, the subject of present investigation) before distribution to the market and consumers. Since this study uses a variety of fishes, a mean moisture content of 77% is used to convert dry-to-wet weight (or vice versa), and the data are given in Table 4.

**Table 4:** Approximate value of dry weight converted from the wet weight recommended by FAO^48^.

Recommended organization	Metal name	Value (wet weight)	Value (dry weight)
FAO	Cr	0.15 to 1.0 mg/kg	0.65–4.35
	Zn	30.0 mg/kg	130.43
	Mn	1	4.35
	Fe	100	434.78
	Co	0.04–0.26	0.17 to 1.13
	Cu	30	130.43
	Se	1	4.35
	Rb	–	–
	Hg	0.5	2.17
	Pb	0.5	2.17
	Ni	80	347.82
	As	1	4.35

The concentration of heavy metals in the dried fish samples is presented in Table 5. Overall, the highest mean concentration was found for Fe, while the As and Ni values were found to be below the detection limit. The concentration of other metals are in the order of Zn > Hg > Cu > Se > Cr > Mn > Co > Rb > Pb. Similar trends in fish species from the Buriganga river in Bangladesh have been previously reported^49^. Similar to the present study, the highest concentration of Fe was also reported in an earlier study by Jezierska and Witeska^50^.

**Table 5:** Concentrations (mg/kg dw) of the determined metals in dried fish collected from Cox’s Bazar. Results are reported as the mean ± SD from triplicate analysis.

Metals	Recommended limit^a^	*H. neherius*	*T. lepturus*	*P. chinensis*	*P. affinis*	*A. mola*	*P. microdon*	*I. megaloptera*	*C. dussumieri*	*L. calcarifer*	*G. chapra*	Mean
Cr	0.65–4.35	7.06 ± 0.06	9.34 ± 0.07	0.42 ± 0.03	3.55 ± 0.04	5.46 ± 0.09	6.67 ± 0.05	9.35 ± 0.03	7.79 ± 0.03	7.39 ± 0.04	12.4 ± 0.0	6.95
Mn	4.35	9.40 ± 0.31	0.33 ± 0.02	0.32 ± 0.01	8.11 ± 0.07	0.32 ± 0.01	1.36 ± 1.80	0.34 ± 0.04	7.45 ± 0.43	0.39 ± 0.07	7.85 ± 0.32	3.59
Fe	434.78	133.5 ± 1.14	191.6 ± 4.2	178.2 ± 4.1	160.8 ± 1.6	148.3 ± 1.1	148.0 ± 1.7	203.0 ± 11.0	172.6 ± 2.3	134.6 ± 4.9	179.0 ± 0.6	164.95
Co	0.17–1.13	0.30 ± 0.02	0.30 ± 0.02	0.31 ± 0.04	2.25 ± 0.48	0.32 ± 0.04	4.32 ± 0.46	0.27 ± 0.01	1.69 ± 0.25	2.65 ± 0.73	0.29 ± 0.01	1.27
Cu	130.43	19.3 ± 0.3	14.5 ± 0.5	0.20 ± 0.12	17.7 ± 0.5	34.7 ± 0.3	10.7 ± 0.2	63.3 ± 2.0	19.4 ± 0.5	19.7 ± 0.6	21.8 ± 0.7	22.12
Zn	130.43	59.2 ± 1.0	50.4 ± 0.8	68.7 ± 0.8	63.5 ± 1.1	43.5 ± 1.5	35.5 ± 0.7	63.7 ± 1.1	51.7 ± 1.4	46.3 ± 1.7	56.8 ± 0.3	53.94
Se	4.35	12.6 ± 0.5	4.73 ± 0.27	0.26 ± 0.03	10.8 ± 0.4	7.77 ± 0.39	5.87 ± 0.79	9.56 ± 0.44	6.77 ± 0.32	11.6 ± 0.6	11.0 ± 0.3	8.09
Rb	–	0.71 ± 0.21	1.52 ± 0.19	0.86 ± 0.06	1.36 ± 0.37	0.86 ± 0.08	0.64 ± 0.09	1.53 ± 0.25	0.74 ± 0.16	0.65 ± 0.10	0.93 ± 0.06	0.98
Hg	2.17	28.7 ± 4.1	48.3 ± 1.0	28.5 ± 0.5	43.6 ± 0.5	30.3 ± 0.8	38.7 ± 2.0	60.2 ± 1.0	37.3 ± 0.9	26.5 ± 0.7	36.5 ± 0.7	37.84
Pb	2.17	0.52 ± 0.02	0.28 ± 0.04	0.001 ± 0.00	0.001 ± 0.00	0.001 ± 0.00	0.001 ± 0.00	0.001 ± 0.00	0.001 ± 0.00	0.001 ± 0.00	0.001 ± 0.00	0.08
Ni	347.82	< 0.24	< 0.24	< 0.24	< 0.24	< 0.24	< 0.24	< 0.24	< 0.24	< 0.240	< 0.24	< 0.24
As	4.35	< 0.41	< 0.41	< 0.41	< 0.41	< 0.41	< 0.41	< 0.41	< 0.41	< 0.41	< 0.41	< 0.41

Concentrations of As and Ni in all analyzed dried fish samples were too low to detect by the adopted EDXRF analytical system. The values quoted herein are the minimum detectable limit (MDL) for these two elements.

^a^Limits from WHO/FAO/MHSAC (Murtala et al.^51^; Ahmed et al.^49^; Alipour et al.^59^).

Chromium (Cr) can exist in several oxidation states from 0 to 6 + . But, the toxicity varying with respect to Cr(III) and Cr(VI)^33^. However, in this study total Cr concentration (both Cr (III) and Cr (VI) together) was determined, ranging from 0.42 ± 0.03 mg/kg in *P. chinensis* to 12.4 ± 0.0 mg/kg in *G. chapra*. According to the FAO, the recommended limits for Cr in fish ranges from 0.65 to 4.35 mg/kg^48^. Only 20% of the fish samples had concentrations within these limits, the other 80% showing values greater than these. This contrasts with a previous report for dried fish sampled in the Cox Bazar region, wherein only 10% of the fish exceeded the recommended limits^49^. With the exception of the lower values of Cr in *P. chinensis* and *P. affinis,* the determined high concentration of Cr in the other species suggests the consumption of these dried fish may induce adverse effect in the human body. The results are in agreement with a number of other fresh fish species studied by Murtala et al.^51^, high levels of Cr being reported in some organs of *H. forskahlii*, *H. bebe* and *C. gariepinus* collected from the Ogun River, Nigeria. Conversely, a lower concentation of Cr has been reported in fish species from the Shitalakkhya River, Dhaka, Bangladesh^52^.

Manganese (Mn) occurs naturally and may reach water bodies through runoff or leaching action via various agricultural activities and anthropogenic sources, agrochemicals in particular. The concentration of Mn measured here in was observed to range between 0.32 ± 0.01 mg/kg in *P. chinensis*/*A. mola* and 9.40 ± 0.31 mg/kg in *H. neherius,* with a mean of 3.59 mg/kg. In *T. lepturus, P. chinensis, A. mola, P. microdon, I. megalopteran and L. calcarifer* Mn was found to be within the recommended limits (4.35 mg/kg) but in all other fish samples (i.e., in 40% of the studied samples) levels exceeded this limit. This differs from values reported by Akter et al.^53^ (with a range from 0.403 to 0.092 mg/kg) and Bashir et al.^54^ (with a range from 0.54 to 79.08 mg/kg). However, present results have been found to be comparable with those of Sivaperumal et al.^55^ in muscle tissue of fish in Indian fish markets (0.14 mg/kg to 3.36 mg/kg).

The recommended limit for Fe in fish is 434.78 mg/kg (Table 5). All samples of the dried fish species analyzed herein showed concentrations in the range 133.5 ± 1.1 to 203.0 ± 11.0 mg/kg, yielding a mean of 165.0 mg/kg, values within the recommended limit. In a related study, Akter et. al.^53^ reported lower concentrations. In this study, Co also showed higher concentrations in *P. affinis, P. microdon, C. dussumieri* and *L. calcarifer* (40% samples) than the recommended limits (0.17–1.13 mg/kg) in all dried fish samples. Fe and Co are essential elements for humans, and their deficiencies can result in skeletal and reproductive abnormalities. Conversely, if the daily intake exceeds the maximum tolerable intake value as discussed in a later section to this paper, then the excess intake of these metals can result in neurological and psychological disorders^56^.

Zn is an essential micronutrient for all organisms. The concentrations of Zn were found to be high in all fish species, but within the FAO^48^ reference limit of 130.0 mg/kg. Similar results have been found in *M. armatus* in an effluent-dominated rivulet in India, as reported by Javed and Usmani^57^ and also in fish from the Buriganga River, Bangladesh^49^. Excess intake of Zn via the consumption of fish having high Zn content can result in nausea, vomiting, loss of appetite, stomach cramps, diarrhea, and headaches.

In this study, only in case of the species *I. megaloptera* (63.3 ± 2.0 mg/kg) show a relatively high concentration of Cu, still within the recommended limit of 130 mg/kg (Table 5). All other fish samples had a nominal concentration within the recommended limit. Akter et al.^53^ reported a very low concentration of Cu (< 1.0 mg/kg) in the dried fish samples that they analyzed. On the other hand, all dried fish samples studied herein showed high concentrations of Se (except in *P. Chinensis*) and Hg than the recommended limits, while all of the fish species show the Pb level within the recommended limit*.* Lower concentrations were reported previously for Se, ranging between 1.68 ± 0.59 and 1.98 ± 0.14 mg/kg, as reported by Ahmed et. al.^49^. Acute exposure to Hg can result in insomnia, neuromuscular changes, headaches and changes in nerve responses^58^. In the case of rubidium (Rb) as reported herein, previous studies have not reported data. Recommended limits for this metal in foodstuffs have also not been reported by any of the advising organizations. Accordingly, we have not found it possible to offer comparisons for this metal.

### Correlation, principal component analysis (PCA) and cluster analysis for source identification

The relationship between metals in the dried fish samples was determined by Pearson’s correlation and the matrix is presented in Table 6. Most metals showed poor correlation except for Hg–Cu (r = 0.605), Fe–Rb (r = 0.739), Fe–Hg (r = 0.754) and Rb–Hg (r = 0.842). Positive correlations between metals in the dried fish samples are potential indications of common pollution sources. The PCA was computed to determine the pollution source(s) of metals in the dried fish samples. The principal components were extracted based on Eigen values > 1, and the results for the total variations are presented in Table 6. The variances for PC1, PC2 and PC3 groups were 90.89, 8.56 and 0.290% respectively (Table 6). Only Fe showed strong factor loading in PC1 suggesting that one particular source may be responsible for Fe contamination of the dried fish samples. The probable source for Fe contamination could be the leaching from the drying pans into the fish samples^60–63^. All other metals showed different anthropogenic and natural sources including atmospheric deposition and the river in which these fish were harvested, studies having shown that rivers in that area receive a high load of anthropogenic and industrial waste effluents (Ahmed et. al.^49^; Akter et. al.^53^; Bashir et al.^64^).

**Table 6:** Pearson’s correlation coefficient between the determined heavy metals and the rotated component matrix for PCA.

	Cr	Mn	Fe	Co	Cu	Zn	Se	Rb	Hg	Pb	PC 1	PC 2	PC 3
Cr	1										−1.68	−0.01	0.050
Mn	0.181	1									−1.84	−0.06	−0.105
Fe	0.252	−0.206	1								6.01	1.89	0.059
Co	−0.136	−0.045	−0.457	1							−1.95	−0.06	−0.017
Cu	0.432	−0.154	0.336	−0.303	1						−1.04	−0.12	0.349
Zn	−0.267	0.290	0.455	−0.583	0.095	1					0.59	0.36	−0.372
Se	0.492	0.524	−0.396	0.053	0.431	−0.034	1				−1.62	−0.04	−0.009
Rb	0.164	−0.152	0.739*	−0.390	0.448	0.434	−0.038	1			−1.97	−0.09	−0.028
Hg	0.372	−0.136	0.754*	−0.103	0.605*	0.200	0.007	0.842**	1		−0.20	0.41	0.105
Pb	0.137	0.329	−0.234	−0.343	−0.134	0.106	0.227	0.019	−0.108	1	−2.02	−0.11	−0.030
Eigenvalues											9.09	0.86	0.029
% of variance											90.89	8.56	0.290
Cumulative %											90.89	99.45	99.74

*Correlation was significant at p < 0.05.

**Correlation was significant at p < 0.01.

**Figure 2:**
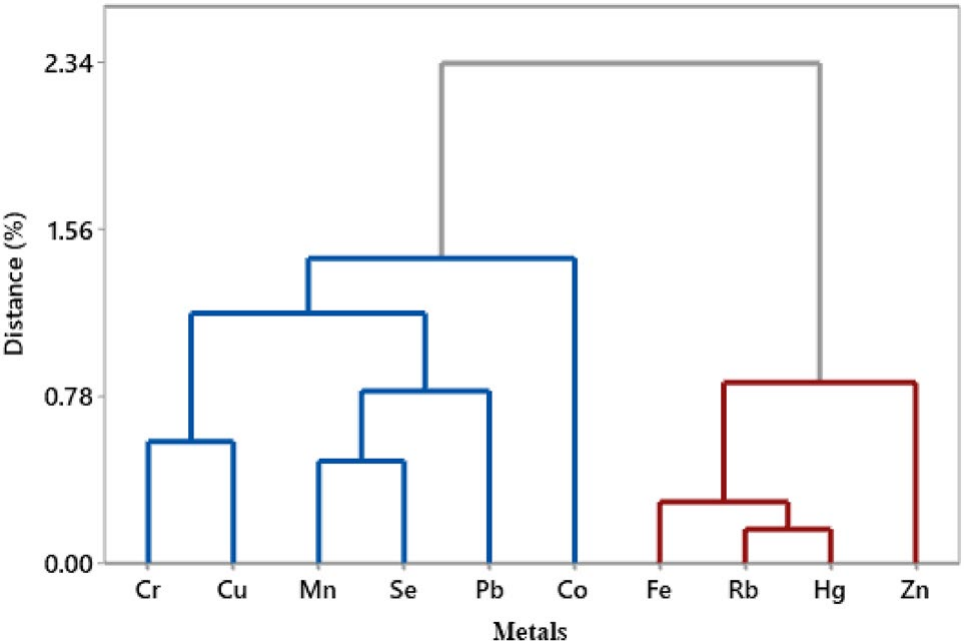
Hierarchical cluster (dendrogram) using the Ward linkage method among the determined metals in dried fish species. The figure was drawn by using SPSS software (Model: IBM SPSS Statistics 21).

The Ward-Linkage method was employed with Euclidean distance, which resulted in three distinct clusters, presented as a hierarchical cluster dendrogram in Fig. 2. Cluster 1 included Cr and Cu that could be sourced from anthropogenic activities like chemical industries, batteries and electrical, fertilizers, textile and fuel. The metals Mn, Se, Pb and Co confined in cluster 2 may arise from the textile industry, fertilizers, pesticides, glass, plastic, alloys and solders and oil spillage from boats/ships in the study area. Finally, Rb and Hg were included in cluster 3, which come from batteries and electrical enterprises, fertilizers, textile, pigments and paints, fuel, medical waste and coal burning.

### Metal concentration factors (MCF) and Metal pollution index (MPI)

**Figure 3:**
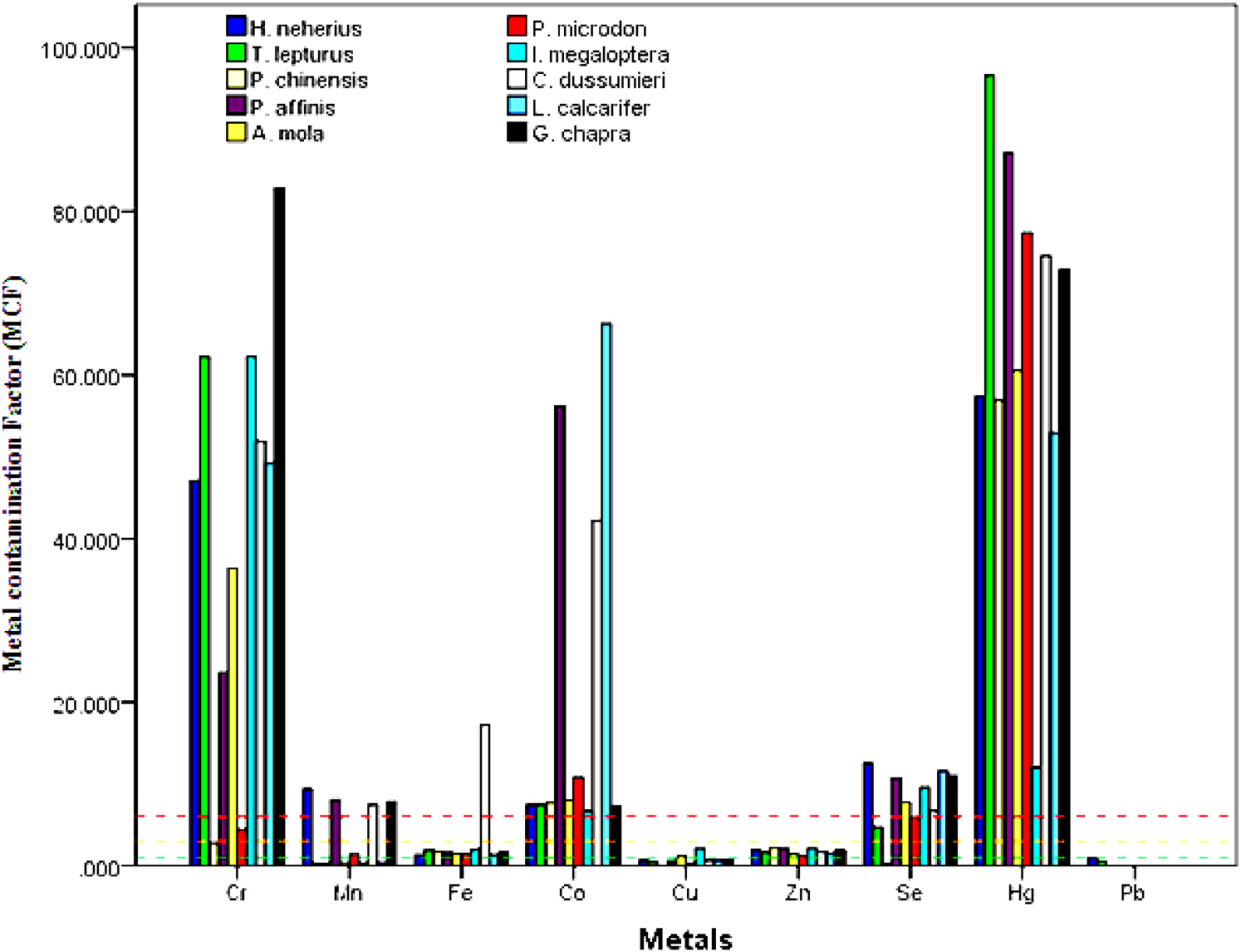
Heavy metal contamination factors in dried fish species. The dashed red lines indicates a very high metal concentration, the dashed orange lines indicates moderate metal concentration while the dashed green lines indicates low metal concentration.

The accumulation or storage of heavy metals by the various fish species was estimated using metal concentration factors (MCF)^47^, acknowledging that the various metal elements have different potentials for being stored in the fish. Potentially storage can achieve levels that may be toxic, hence the variation between recommended permissible levels. Expressing metal concentration as a determinant in fish in terms of a factor that is indicative of permissible limits can enable the fish species to be ranked according to their tendency to accumulate a particular metal. The MCF values for the studied metals in the different dried fish species are presented in Fig. 3. All fish species with the exception of *H. neherius* showed a low accumulation of Pb (tending towards moderate in *H. neherius*), all species showing moderate levels for Zn, and a very high accumulation for Co, Hg and Se. However, for Cr, only *P. microdon* was accumulated moderately, all others being very high. Similarly, *P. microdon* also showed moderate accumulation for Mn, while *T. leptutus*, *P. chinensis*, *A. mola*, *I. megaloptera* and *L. calcarifer* were low while other fish species were represented in the following order *H. neherius* (9.4) > *P. affinis* (8.11) > *G. chapra* (7.85) > *C. dussumieri* (7.45), showing high accumulation of Mn. All fish species showed moderate accumulation for Fe except for *C. dussumieri* (17.26) which was very high. Only *A*. mola (1.15) and *I. megaloptera* (2.11) showed moderate accumulation of Cu, while others were low. Similar to this study, high MCF for metals have been reported for a number of fish species collected in lake Oguta in Nigeria^47^.

**Figure 4:**
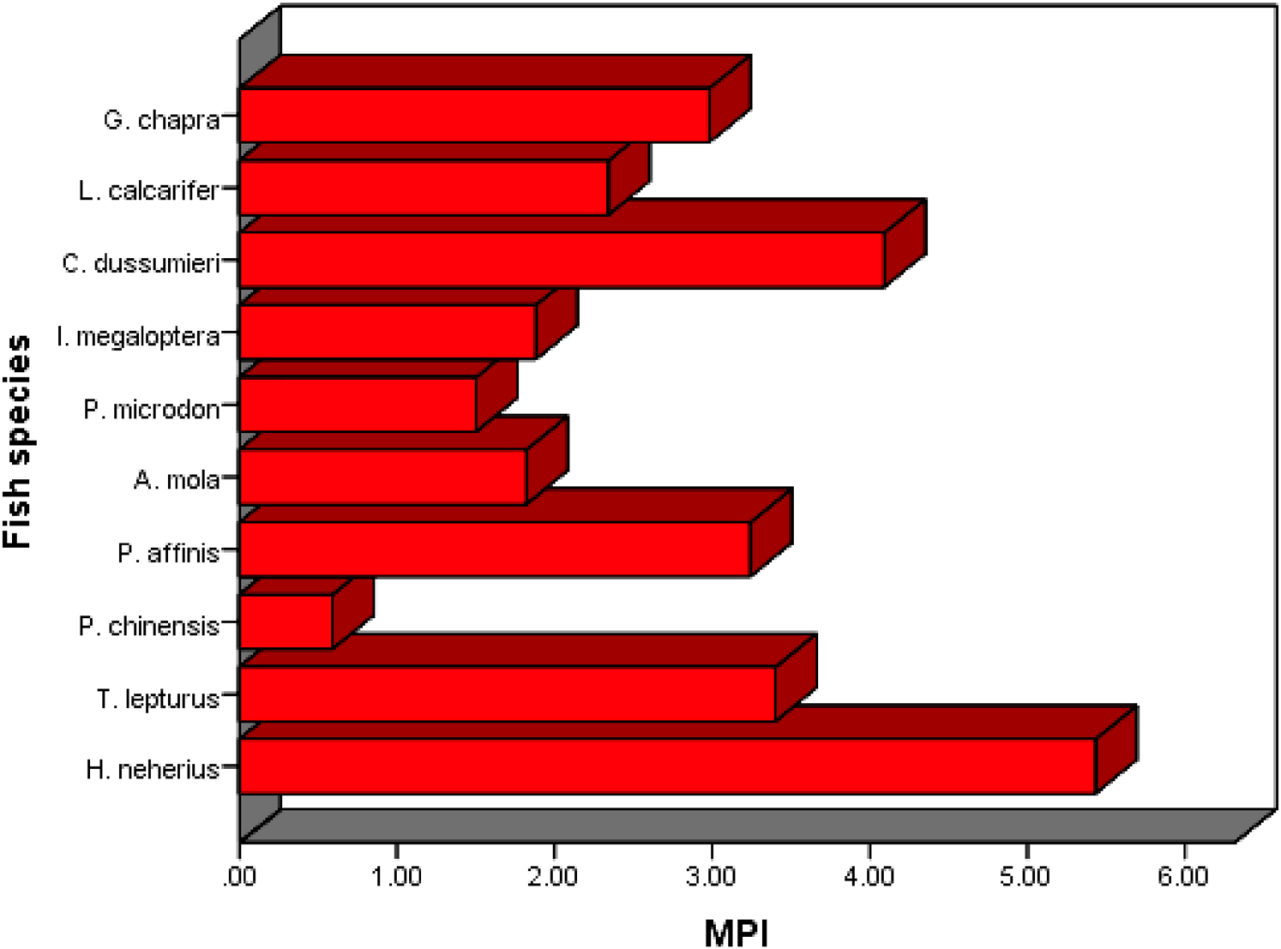
Metal pollution index for the dried fish species presently investigated.

The extent of heavy metal load in all of the dried fish samples, measured with respect to reference or standard values, was estimated using the metal pollution index (MPI), the results being presented in Fig. 4. The highest values was found in *H. neherius* while the least was that in *P. chinensis*. All fish species showed moderate to high pollution with an exception for *P. chinensis*. Accordingly, consuming these dried fishes may in some cases pose non-negligible health implications to humans. The results show considerable difference compared to the study of Akter et al.^53^ on dried fish species from Cox’s Bazaar, noting that, Akter et al.^53^ analyzed dried fish species that with the exception of *H. neherius* were different from those studied herein.

### Estimated daily intake of heavy metals

**Figure 5:**
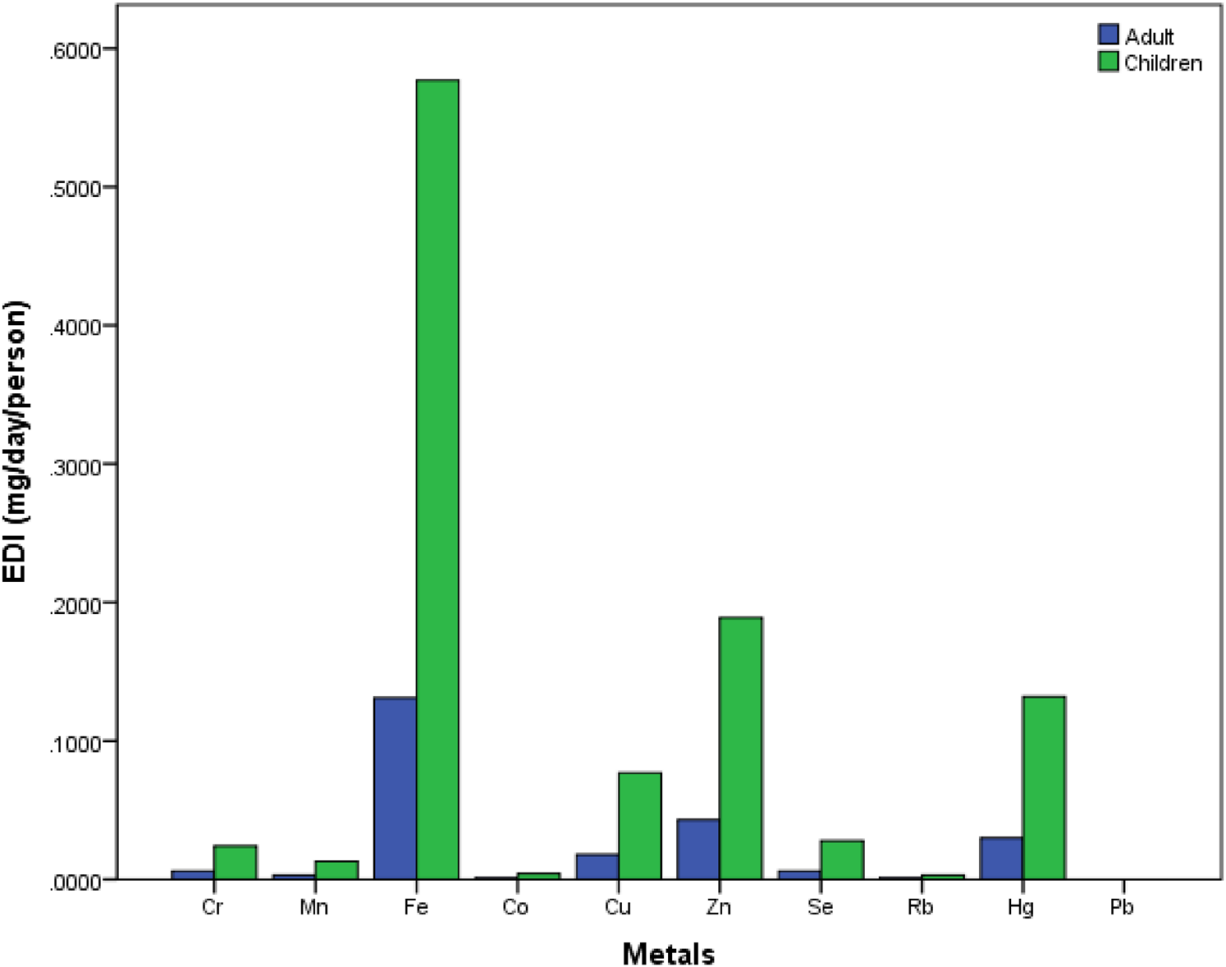
Estimated daily intake (EDI) of metals for adults and children via the consumption of dried fish.

The estimated daily intake (EDI) was computed for the various metals in consuming the dried fish species, comparing the EDI for adults and children both in the study area as well as for other locations, these fish being commercially sold in different markets throughout the country. The results are presented in Fig. 5. Generally, a higher intake of heavy metals is shown for children. Fe showed the highest EDI with 0.131 mg/day/kg for an adult and 0.577 mg/day/kg for children. The least intake was for Rb with 0.001 mg/day/person for adults and 0.003 mg/day/kg for children. The calculated EDI for the studied metals show a low value when compared with the maximum tolerable intake (MTDI) value, noting that the MTDI is set as a safe limit for lifetime exposure^65^. Earlier studies in Bangladesh by Ali et. al.^66^ obtained similar results to the present work, no concern being found in respect of risk to health. However, an increase in the frequency of fish consumption or metal contamination within the fish may lead to adverse health effects to the Bangladeshi population^67^.

### Non-carcinogenic and carcinogenic health risks

The non-carcinogenic target hazard quotient (THQ) and hazard index (HI), and carcinogenic health risks are presented in Tables 7 and 8. In reference to single elements, THQ and HI > 1 indicate potential adverse health effects^68^. The calculated THQ were less than 1 for all the element except for Hg to children for the following fish species *H. neherius, T. lepturus, P. affinis, A. mola, P. microdon, I. megaloptera, C dussumieri and G. chapra*. According to the WHO^69^, exposing children to high Hg concentration can cause kidney damage, gastrointestinal tract infection and skin acrodynia^69^. This is due to the high THQ of Hg in the fish samples. Therefore, control on consumption should be practiced for children. The HI for adults is generally shown to be less than 1 for all studies fish species while children showed non-carcinogenic health risks for the following fish species: *H. neherius, T. lepturus, P. affinis, A. mola, P. microdon, I. megaloptera, C. dussumieri and G. chapra.*

**Table 7:** Calculated THQ and HI for two selected age groups, adults (Ad) and children (Ch).

Species	THQ (Cr)		THQ (Co)		THQ (Cu)		THQ (Zn)		THQ (Hg)		THQ (Pb)		THQ (Mn)		HI	
	Ad	Ch	Ad	Ch	Ad	Ch	Ad	Ch	Ad	Ch	Ad	Ch	Ad	Ch	Ad	Ch
*H. neherius*	0.002	0.008	1.19E−05	5.25 E−05	0.001	0.002	1.57 E−04	6.91 E−04	0.228	1.005	1.03 E−04	4.55 E−04	1.62E−04	7.15E−04	0.332	1.02
*T. lepturus*	0.002	0.011	1.19E−05	5.25 E−05	0.001	0.001	1.33 E−04	5.88 E−04	0.383	1.691	5.55 E−05	2.45 E−04	5.69E−06	2.51E−05	0.386	1.704
*P.s chinensis*	0.000	0.000	1.23E−05	5.43 E−05	7.93E−06	1.75 E−05	1.82 E−04	8.02 E−04	0.226	0.996	1.98E−07	8.75 E−06	5.52E−06	2.43E−05	0.226	0.996
*P.s affinis*	0.001	0.004	8.92E−05	3.94 E−04	0.001	0.002	1.68 E−04	7.41 E−04	0.346	1.526	1.98E−07	8.75 E−06	1.39E−04	6.17 E−04	0.348	1.533
*A. mola*	0.001	0.006	1.27 E−05	5.6 E−05	0.001	0.003	1.15 E−04	5.08 E−04	0.239	1.059	1.98E−07	8.75 E−06	5.52E−06	2.43E−05	0.241	1.068
*P. microdon*	0.002	0.008	1.71 E−04	7.56 E−04	4.23 E−04	0.001	9.39E−05	4.14 E−04	0.307	1.354	1.98E−07	8.75 E−06	2.34E−05	1.03 E−04	0.309	1.364
*I.megalopteran*	0.002	0.011	1.07E−05	4.73 E−05	0.003	0.012	1.68 E−04	7.43 E−04	0.477	2.106	1.98E−07	8.75 E−06	5.86E−06	2.59E−05	0.482	2.129
*C. dussumieri*	0.002	0.009	6.69E−05	2.96 E−04	0.001	0.002	1.37 E−04	6.03 E−04	0.296	1.305	1.98E−07	8.75 E−06	1.28E−04	5.67 E−04	0.299	2.129
*L. calcarifer*	0.002	0.009	1.05 E−05	4.64 E−04	0.001	0.002	1.22 E−04	5.4 E−04	0.209	0.926	1.98–07	8.75 E−06	6.72E−06	2.97E−05	0.212	0.938
*G. chapra*	0.003	0.015	1.15E−05	5.08 E−05	0.001	0.002	1.5 E−04	6.63 E−04	0.289	1.276	1.98E−07	8.75 E−06	1.35E−04	5.97 E−04	0.293	1.294

**Table 8:** Computed cancer risks (CR) from the two selected age groups, adults and children.

Species	CR (Cr)		CR (Pb)	
	Adult	Children	Adult	Children
*H. neherius*	2.79E−06	1.24 E−05	3.50E−09	1.55E−08
*T. lepturus*	3.70E−06	1.63 E−05	1.89E−09	8.33E−09
*P. chinensis*	1.67E−07	7.35 E−06	6.74E−12	2.98E−11
*P. affinis*	1.41E−06	6.21E−06	6.74E−12	2.98E−11
*A. mola*	2.16E−06	9.56 E−05	6.74E−12	2.98E−11
*P. microdon*	2.64E−06	1.17E−05	6.74E−12	2.98E−11
*I. megalopteran*	3.71E−06	1.64E−05	6.74E−12	2.98E−11
*C mieri*	3.08E−06	1.36E−05	6.74E−12	2.98E−11
*L. calcarifer*	2.93E−06	1.29E−05	6.74E−12	2.98E−11
*G. chapra*	4.93E−06	2.18E−05	6.74E−12	2.98E−11

The carcinogenic risk was only assessed for Cr and Pb in the various fish species due to either the lack of a slope factor for the other studied metals or non-detection of other carcinogenic metals. The acceptable limit for CR lies between 10^–6^ to 10^–4^. For both adults and children, the obtained results show all estimated values for Cr and Pb for all fish species to be within the safe limiting range. Results generally indicate Pb to be a predominant contaminant, with an associated relatively higher cancer risk compared to Cr, a result agreeing with that obtained by Enyoh and Isiuku^68^.

## Conclusions

This work presents the first comprehensive data on essential and non-essential metals in ten popularly consumed species of dried fish, all collected from the local markets of Cox’s Bazar region. A state-of-the-art, non-destructive, low cost, simple and rapid EDXRF technique was employed to determine the metal concentrations in the studied fish samples. In almost all samples, the studied elements were found to vary in concentration, all fish species showing moderate-to-high pollution with the exception of *P. chinensis*. The probable source of contamination has been suggested to arise from leaching from the drying pans into the fish, atmospheric deposition and the river/marine waters in which these fish were harvested. The consumption of these fish show a greater non-carcinogenic risk for children than for adults. The carcinogenic risk was found to be within the safety limit recommended by the various international advisory organizations. Periodic monitoring of trace metals in edible aquatic organisms and fish is recommended in seeking to avoid unexpected health hazards.
